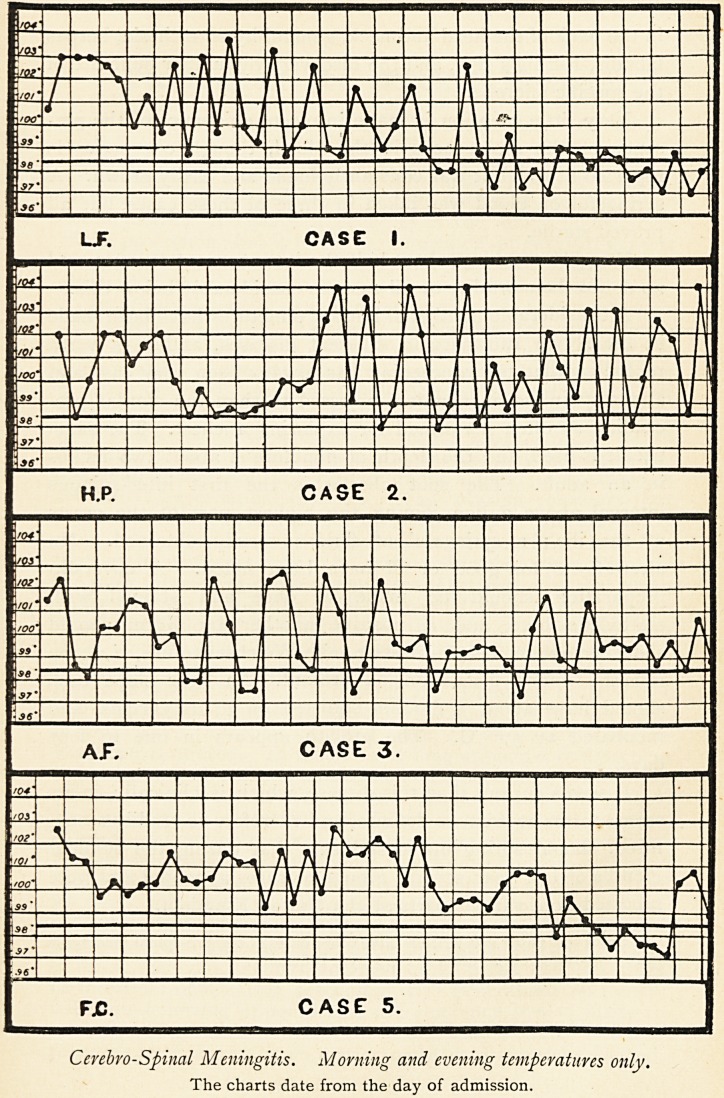# Six Cases of Cerebro-Spinal Meningitis

**Published:** 1901-03

**Authors:** E. H. Edwards Stack

**Affiliations:** House Physician, Royal Infirmary, Bristol.


### SIX CASES OF CEREBRO-SPINAL MENINGITIS.

BY

E. H. Edwards Stack, M.B., F.R.C.S.,

*House Physician, Royal Infirmary, Bristol.*

---

These cases were all patients in the Royal Infirmary, and I wish to thank the physicians under whose care they were for permission to publish these notes.

The first five were admitted within a few weeks of each other last summer; but as they came, with two exceptions, from widely different districts, they can scarcely be said to constitute an epidemic. Cases 1 and 2 were father and daughter, living in the same house. The latter was the first case. The disease ran a short and mild course, and was not suspected, but there seems to be very little doubt that they were both due to the same micro-organism.

The sixth case occurred in January of this year, and is included as it is the most typical of the series.

CASE I.—L. F., aged 17, a girl. Admitted, under Dr. Shingleton Smith, on May 5th, 1900; discharged June 14th, recovered. Previous history unimportant. Five days ago patient was attacked with pains in the head, back, and limbs; a few days later she was "raving with pains in her head."

On admission: patient was thin, very deaf, and having headache; heart, lungs and abdomen natural; eyes and fundi natural. May 12th.—Temperature very erratic, between 100° and 103°; since admision complains of pain in back of head and neck; general hyperæsthesia. May 20th.—No physical signs in chest or abdomen have developed; knee-jerks natural; pneumonia can be excluded, and Widal has twice been negative; neck is stiff; temperature quieting down. June 14th.—She has made a slow and steady recovery since the last note, and is going out to-day.

This case was a daughter of A. F. (Case 3), aged 45, who came in a week before she was discharged, and who died of cerebro-spinal meningitis. While at the Infirmary this disease was not suspected, and neither lumbar puncture nor Kernig's sign[1] was investigated.

CASE 2.—H. P., aged 16, a boy. Admitted, under Dr. Shaw, on May 25th, 1900; death on June 19th, 1900. Three weeks ago, while carrying a bar of iron, fell and struck the back of his neck on a wooden block; he did not notice any ill effects of this for two days, then he developed a headache, which continued "on and off" till admission; during this time he remained in bed; two days previous to admission he vomited once. His temperature had been taken since 11th of May, and had been very irregular, ranging from normal to 104°.

On admission: only complains of his headache; well-nourished boy, does not look ill; pulse 80, respiration 22, temperature 101.2°; appetite poor, tongue furred; no herpes labialis, no rash; lungs, heart, and abdomen are natural; eye movements and fundus natural. June 1st.—Spine is tender to movement and percussion from the third to the sixth dorsal, and held very rigid in this region; the head is becoming retracted. June 6th.—Head retracted to a right angle; mentally very clear, and looking very unlike meningitis; knee-jerks have become much more active than on admission; Kernig's symptom well marked; cannot now sit up without support. June 9th.—Lumbar puncture gave serum slightly opalescent; fresh coverslip preparations

[1] Kernig's sign is the inability to extend the patient's leg on the thigh while the thigh is at right angles to the body, and is due to a contraction of the flexor muscles; in a marked case the leg can only be brought as far as a right angle with the thigh.

showed the presence of numerous diplococci, many of them in leucocytes, which stained easily with methylene blue and did not retain their stain when treated by Gram's method; agar tubes and blood agar tubes were stroked with the fluid, and four days later showed a number of small round discrete colonies with the same morphological appearance and staining properties; the headache remained the same after puncture as before (about three drachms were removed). June 12th.—Boy has gradually become weaker and more apathetic, he can just understand enough now to put out his tongue when told to; head is retracted beyond the right angle; spine remains straight; there is no change in the fundus; there has been no herpes or rash of any sort; he is passing everything under him. June 19th.—He died this morning. Yesterday he was fed with a nasal tube. The eyes were wandering independently.

P.M.E.—Basal sero-purulent meningitis. The cord did not appear to be affected.

CASE 3.—A. F., aged 45 (father of Case 1). Admitted, under Dr. Shaw, June 9th, 1900. Five years previous to admission he was in the same ward under Dr. Shaw, for headaches, for which no definite cause could be assigned. About a month ago he began to complain of "headache and weakness," and he had to cease work, he felt "very weary." Two weeks ago pain in the head got worse, and he began to "wander"; vomiting started, and with it diarrhœa, and for a week he had been passing everything under him.

On admission: patient lies on his back in a drowsy state, and looks like a bad case of enteric in the fourth week, muttering to himself about his work, and taking no notice of anything; pulse feeble, 124; respiration shallow, 20; temperature 101°. There is nothing noteworthy in the chest or abdomen; eye movements are natural, and so is the fundus on both sides. He is somewhat incoherent, but on commanding his attention he understands what is said, and answers with correct words which are rather difficult to recognise; he continues to pass everything under him; there is no retraction of the head, but he objects to his head being moved; urine drawn off with a catheter contains albumen and a few granular casts. July 4th.—Patient has scarcely changed in any way since admission; he is getting thinner; he still lies semi-conscious and mutters; temperature is very irregular, between normal and 103°, following no definite course; there seems to be a little weakness in the right leg, and it has been slightly œdematous. Kernig's sign is marked, and the flexors of arms are resistant; there are no changes anywhere in sensation; several Widal reactions have been negative. September 4th.—No certain diagnosis has been arrived at. Six times has a lumbar puncture been made, the first was on the 4th of July: the fluid is always clear. In one of these a micro-organism was found, which had grown on an agar slope, and this reacted to stains as the diplococcus intracellularis does, and therefore the case is supposed to be one of very chronic cerebro-spinal meningitis. He is slowly sinking week by week. 10th September he died.

P.M.E. showed basal meningitis in the subarachnoid space, and a few flakes on the surface of the cervical region of the spinal cord.

CASE 4.—E. E. R., aged 9, a girl. Admitted, under Dr. Waldo, on June 11th, 1900. On June 10th she went for "too long a walk," and "it was very hot." She vomited when she got home, and next morning refused her breakfast and complained of pains in her legs.

On admission: she looks like a cerebral case; turns about in bed, chatters sense, generally keeps her knees drawn up, and complains of pains in the legs; she has sordes and a furred tongue; her head aches very much, and she complains of great pain in the back of her neck when her head is moved; lungs, heart, and abdomen natural; pulse 100, respiration 36, temperature 100°; eyes—no strabismus, left optic disc shows veins fuller than natural, right not seen. June 15th.—Paralysis of left external rectus; herpes at left angle of the mouth, still conscious, and not worse; complains of pain in the abdomen; Widal negative; no vomiting; Kernig's sign well marked. June 19th.—There is now no strabismus or weakness of the recently paralysed muscle, but she has double optic neuritis; no retraction of the head; still very great headache. June 30th.—Getting weaker, and very thin; incontinence of urine since the 24th, still shrieks occasionally with headache; she had typical Cheyne-Stokes' respiration during sleep for two nights; still counts fingers properly. July 17th.—Has begun to improve, not noisy now; temperature, which has been ranging between 99° and 103° in a very irregular manner, has now rather suddenly become subnormal. For the first three weeks the temperature generally moved through two or three degrees every day, sometimes rising in the evening and at others in the morning; since July 4th it has been steadier, from 100° to 101°, till it fell on the 14th from 101° to 97°. Optic neuritis still present. August 2nd.—A lumbar puncture was made, but it was sterile; Kernig's symptom has disappeared; sits up now. Sept. 2nd.—Walks about, weak still; bright and intelligent and gaining flesh; and going home. Jan. 12th, 1901.—Is quite well.

Case 5.—F. C., aged 7. Admitted, under Dr. Shingleton Smith, on June 15th, 1900. Three weeks ago the child was knocked down by a bicycle, but was not injured in any way; ten days ago, suddenly, convulsions, and ever since "all of a work"; remained "unconscious" till day before admission; sent in as meningitis.

On admission: thin and irritable, looks very ill; keeps chattering coherent nonsense; lies on side or back, and always with knees flexed; no vomiting; seems to have pain in ears and legs, and to be tender everywhere, and hyperæsthetic; there is no strabismus or any paralysis; lungs, heart, and abdomen natural; trace of albumen in urine; knee-jerks increased, no ankle-clonus, plantar reflex flexor type; Kernig's sign well marked, and also rigidity of arm flexors; pupils normal, and react to light, optic disc slightly choked; no optic neuritis; Widal's reaction negative. June 19th.—Lumbar puncture on the 16th, serum opalescent, and a coverslip preparation showed diplococci intra- and extra-cellular, a growth appeared to-day on an agar tube which had been inoculated, this was composed of diplococci which stained with methylene blue, but were decolourised by Gram's method; herpes labialis, which appeared on the 16th, also showed a diplococcus with the same characters; very drowsy, and complaining of pain all over. June 26th.—Lumbar puncture, tube inoculated remained sterile. June 30th.—Nasal feeding; more delirious; head retracted; passes everything in bed; getting much thinner. July 1st.—Looks as though recovery was impossible. July 7th.—Very much better, talks rationally; very weak, no stiffness of neck. Temperature from admission till July 2nd was very irregular, from 100° to 102°, after that date it became gradually normal. July 19th.—Patient is well, but he was very thin and weak for a long time.

Case 6.—M. W., aged 21, a woman. Admitted, under Dr. Prowse, on January 15th, 1901. Death on January 26th. History of present illness was—pains in the head, followed by pain in all the limbs, two weeks ago.

On admission: pain occasionally excruciating in head and back of neck; neck is stiff, but there is no retraction; face flushed, and herpes is present about the mouth; very torpid, she has been deaf since childhood, but is much more so now; sight, smell, and taste natural; Kernig's sign well marked: no rash; arms are quite flaccid. Jan. 17th.—Now slight retraction of head; knee-jerk absent on right side; eyes natural, no optic neuritis; lumbar puncture showed turbid serum with intracellular diplococci; complains very much of diplopia; no albumin. Jan. 18th.—Suddenly got very ill, looking as though she were about to die, and became semi-comatose. Jan. 19th.—Again sensible. Jan. 22nd.—Neither knee-jerk obtainable; urine readily reduces Fehling's solution. Jan. 24th.—Quite blind since yesterday; left ext. rectus paralysed, and fundus natural; neck still very painful. Vomited once. Jan. 25th.—Urine contains 1 per cent. of albumin, no sugar, urea 4 per cent.; neck better, less deaf and more sensible. Jan. 26.—Without warning she suddenly died. Three lumbar punctures all gave typical growths of the specific organism. Coverslip preparation of blood from a vein showed no micro-organims, and an agar tube inoculated remained sterile. The temperature has been irregular, from 99° to 103°.

P.M.E.—The base of the brain from the optic nerves backwards was thickly covered with lymph, also the under surface of the cerebellum. The cord had œdematous lymph as far down as the dorsal region lying on the pia mater. No excess of fluid in the ventricles, and no lymph on the convexity of the brain.

On looking over old and recent accounts of this disease, there seems to be no doubt that it must be commoner than is supposed, and that a number of cases have occurred which have not been diagnosed. Sometimes it appears as a mild attack which has passed as febricula, a bilious attack, herpetic fever, etc., and on the other hand it is probable that many cases which were called simple or posterior basic meningitis had the same etiology. The disease used to be considered due to the diplococcus pneumoniæ, and it seems certain that this microbe is occasionally the exciting cause. Others have described cases of streptococcal origin. One, therefore, has to consider that practically the same clinical disease is due to several different micro-organisms. In such a complicated and often so definite a condition this is curious; and should the question of treatment by a specific antitoxin arise it would certainly be important that a differential diagnosis should be established, and therefore lumbar puncture will not only be interesting but useful.

The most constant conditions found on examination seem to be: an irregular temperature not assignable to other causes, pain

*Cerebro-Spinal Meningitis. Morning and evening temperatures only.*
The charts date from the day of admission.

in the back of the head and neck, general hyperæsthesia, herpes labialis, Kernig's sign, and the discovery by lumbar puncture of the specific microbe.

Very little mention is made in recent epidemics of the presence of the diplococcus in the blood, though one series is reported in which it was constantly found and easily grown. A syringeful of blood was taken in three of these cases, but all proved sterile.

Roughly speaking, about half the known cases recover, and no doubt the mortality of all cases is considerably below this.

A considerable number of lumbar punctures have been made in the Royal Infirmary in different diseases, and it is worth recording that the proceeding has always been very easy and almost painless, in fact less objected to than the similar proceeding so often performed for exploring a chest. The same needle is used, and the depth of puncture is about two inches in an adult. The spot chosen is the first inter-spinous interval above a line joining the highest part of the crests of the ilia; a syringeful of fluid is easily obtained. No after-effects for good or ill have been noted. A coverslip preparation should be made at once and stained with methylene blue, and if positive another by Gram should show complete decolourisation, if Weichselbaum's, *i.e.* the microbe under discussion, is present. An agar slope tube should have about a drachm squirted on to its surface, and incubated at 37° C. The growth appears in one to four days.

It seems curious that this disease, which is clinically, in its severe forms at least, so definite, and therefore so readily recognised, should spring up so suddenly that during the summer months of last year several groups of cases appeared and were reported from various parts of the United Kingdom.

Since this paper was written three other cases of cerebro-spinal meningitis have been under observation at the Royal Infirmary.

## Figures and Tables

**Figure f1:**